# Risk profiling of schistosomiasis using remote sensing: approaches, challenges and outlook

**DOI:** 10.1186/s13071-015-0732-6

**Published:** 2015-03-17

**Authors:** Yvonne Walz, Martin Wegmann, Stefan Dech, Giovanna Raso, Jürg Utzinger

**Affiliations:** Department of Remote Sensing, Institute for Geography and Geology, University of Würzburg, Würzburg, Germany; United Nations University – Institute for Environment and Human Security, Bonn, Germany; German Remote Sensing Data Centre, German Aerospace Centre, Oberpfaffenhofen, Germany; Department of Epidemiology and Public Health, Swiss Tropical and Public Health Institute, Basel, Switzerland; University of Basel, Basel, Switzerland

**Keywords:** Ecology, Epidemiology, Geographical information system, Intermediate host snail, Remote sensing, Risk profiling, Scale, Schistosomiasis, Spatial modelling

## Abstract

**Background:**

Schistosomiasis is a water-based disease that affects an estimated 250 million people, mainly in sub-Saharan Africa. The transmission of schistosomiasis is spatially and temporally restricted to freshwater bodies that contain schistosome cercariae released from specific snails that act as intermediate hosts. Our objective was to assess the contribution of remote sensing applications and to identify remaining challenges in its optimal application for schistosomiasis risk profiling in order to support public health authorities to better target control interventions.

**Methods:**

We reviewed the literature (i) to deepen our understanding of the ecology and the epidemiology of schistosomiasis, placing particular emphasis on remote sensing; and (ii) to fill an identified gap, namely interdisciplinary research that bridges different strands of scientific inquiry to enhance spatially explicit risk profiling. As a first step, we reviewed key factors that govern schistosomiasis risk. Secondly, we examined remote sensing data and variables that have been used for risk profiling of schistosomiasis. Thirdly, the linkage between the ecological consequence of environmental conditions and the respective measure of remote sensing data were synthesised.

**Results:**

We found that the potential of remote sensing data for spatial risk profiling of schistosomiasis is – in principle – far greater than explored thus far. Importantly though, the application of remote sensing data requires a tailored approach that must be optimised by selecting specific remote sensing variables, considering the appropriate scale of observation and modelling within ecozones. Interestingly, prior studies that linked prevalence of *Schistosoma* infection to remotely sensed data did not reflect that there is a spatial gap between the parasite and intermediate host snail habitats where disease transmission occurs, and the location (community or school) where prevalence measures are usually derived from.

**Conclusions:**

Our findings imply that the potential of remote sensing data for risk profiling of schistosomiasis and other neglected tropical diseases has yet to be fully exploited.

## Background

**Figure 1:**
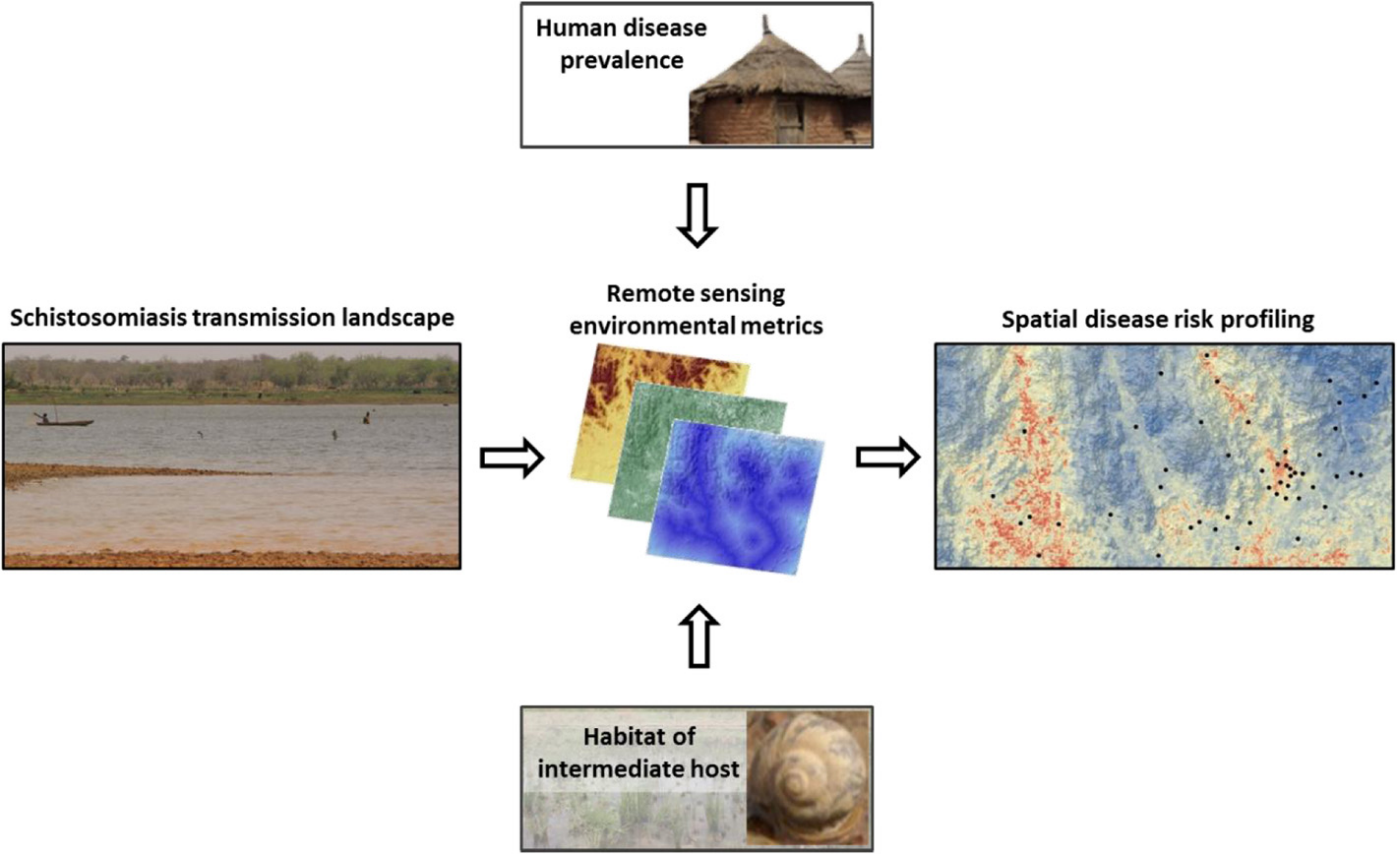
**Conceptual framework of schistosomiasis risk profiling using remote sensing data.** Remote sensing data measure environmental conditions that describe landscape parameters relevant for disease transmission. The information of suitable habitat conditions for intermediate host snails and *Schistosoma* parasites or survey measurements of human disease prevalence provide the reference of remotely sensed environmental metrics. Based on this established relation, remotely sensed environmental conditions can predict the risk of disease transmission in geographical space.

Schistosomiasis is a parasitic disease of humans and animals caused by blood flukes of the genus *Schistosoma*. From a public health perspective, schistosomiasis is the most important water-based disease [1]. Global statistics suggest that an estimated 779 million people are at risk of schistosomiasis [1], about 250 million people are currently infected, mainly in sub-Saharan Africa [2], and the global burden of schistosomiasis is 3.3 million disability-adjusted life years (DALYs) [3]. Transmission of schistosomiasis is spatially and temporally restricted to freshwater bodies inhabited by specific intermediate host snails, which act as disease vectors by shedding cercariae. Schistosomiasis is an environmental disease and remote sensing technologies can deepen our understanding of environmental drivers in relation to the spatial and temporal distribution [4]. Figure 1 illustrates the conceptual framework adopted here for spatially explicit schistosomiasis risk profiling using remote sensing data.

Over the past 30 years, the use of remote sensing data and techniques in mapping environment-related diseases, including schistosomiasis, has increased considerably [5]. The wealth of scientific literature contains a set of case studies that investigated the usefulness of various satellite data and variables with their spatial and temporal properties for selected geographical regions of the world and in relation to specific diseases or disease agents such as intermediate hosts, parasites or vectors [5-12]. Since those agents have specific requirements regarding climate, vegetation, soil and other edaphic factors, and are sensitive to changes therein, remote sensing can be used to determine the living conditions of these agents and predict potential distributions of their habitats (for definition, see Table 1) [13].

**Table 1:** **Important terms and definitions employed in the current review**

**Term**	**Definition**
Risk	Defined as effect of uncertainty on objectives (International Organization of Standardization (ISO) 31000: Risk management) and implies a future event with an uncertainty if and how the entity of interest is affected by a certain phenomenon. For this research, risk is defined as probability of humans to become infected with the parasite.
Environment	In a medical sense, integrates all factors external to humans but interacts with them, e.g. physical, biological, social and cultural environment [14]. With respect to the parasite and snail species, environment includes biotic and abiotic phenomena surrounding and potentially interacting with the organisms [15].
Habitat	Description of a physical place (i.e. a geographical space), at a particular scale of space and time, where an organism either actually or potentially lives [15].

It is important to note that there is a complex linkage between ecological habitat conditions of disease-related parasites and intermediate host snails, relevant measurements of remote sensing data and infection of humans, which has not yet been analysed and reviewed by different groups that bridge professional expertise of the respective disciplines [16]. For example, it has never been specified what different remote sensing measurements (e.g. normalized difference vegetation index (NDVI)) exactly detect with regard to the ecological context of schistosomiasis transmission. However, a detailed understanding of the respective measurements from each discipline against the contextual background of disease transmission depicted in Figure 1 is necessary to optimise its application and improve spatial risk (for definition, see Table 1) assessment and prediction.

The aim of this review was to build a bridge between the knowledge gathered between different disciplines; namely, epidemiology and disease ecology on the one hand and remote sensing measurements used for risk profiling on the other hand. Schistosomiasis serves as the example to connect the different strands of scientific inquiry. We review factors that might govern the transmission of schistosomiasis, placing particular attention to remote sensing applications for risk profiling. In this regard, our review builds upon the work of Simoonga et al. [11] and is complemented with a detailed analysis of remote sensors, sensor combinations and specific variables that have been used to date for schistosomiasis risk profiling.

### Factors that govern schistosomiasis risk

Risk of schistosomiasis is defined as the probability of humans becoming infected with *Schistosoma* during contact with infested freshwater bodies. The rate of human infection is fundamentally governed by suitable conditions for disease transmission from human to human, which integrates factors that determine the parasite life cycle, its intermediate host snail and its definitive human hosts, as illustrated by King [17]. The impact of relevant disease transmission factors on the ecology of the parasite, intermediate host snail and human host infection are summarised in Table 2 and described in more detail in the following paragraphs.

**Table 2:** **Overview of parasite-, snail- and human-related factors that modify, retain or intensify the cycle of schistosomiasis transmission**

**Parasite-related factors**	**Effect on schistosomiasis transmission**	**Reference(s)**
Temperature	Length of prepatent period; activity, survival and infection rate of free-living stages of the parasite	[18,19]
Water flow velocity	Passive transport of parasites in flowing water determines cercarial density	[20]
Predators	Fish and carnivorous invertebrates reduce parasite population as natural predators	[20,21]
Sunlight	Stimulation of cercarial shedding	[19]
Pathogenicity	Different strains of *S. mansoni* and *S. haematobium* result in geographical variations of disease severity	[22]
Species	Different efficiency in identifying and infecting snails	[19]
**Snail-related factors**	**Effect on schistosomiasis transmission**	**Reference(s)**
Water temperature	Fecundity, mortality and rate of reproduction	[18,23-26]
Water flow velocity	Flow velocity >0.3 m/s may dislodge and swep away snails	[24,27,28]
Vegetation	Food supply, surface to crawl and deposit egg masses; increase of dissolved oxygen	[23]
Substratum	Nature of substratum is related to snail abundance	[23,24]
Water depth	Snails generally found in shallow water near the margins of their habitats; below 1.5-2 m, snails have little importance for the transmission of schistosomiasis	[29]
Fluctuations of water level	Permanence of available habitats determines the distribution patterns of snails	[23,24]
Rainfall	Creation of temporary snail habitats; increase of water flow velocity; supports contamination of water and passively transports snails when rains are heavy	[20,24,27]
Turbidity	Turbidity can impact the reproduction cycle	[24,30]
Water chemistry/quality	Low pH, refuse from factories directly harm snails; high abundance where water is polluted with human excrements	[23,24,27,31]
Sunlight	Completely shaded pools provide unsuitable habitat and activity of snails is high in direct sunlight	[23]
Predators/pathogens	Natural predators, parasites and pathogens may limit the abundance of snails	[23]
Species	Variation of susceptibility to parasite and efficiency to produce cercariae	[27,32]
**Human-related factors**	**Effect on schistosomiasis transmission**	**Reference(s)**
Water contact behaviour	Exposure of the skin to parasite infested water is the prerequisite for human infection	[33]
Hygiene	Contamination of water due to excrements of infected humans in or aside water	[20,34,35]
Gender	Relationship between gender and risk of infection is culturally variable and a determinant of water contact activities	[35-37]
Age	Highest risk for children as consequence of degree of exposure and low level of immunity	[33,35,38]
Immunity	Resistance to reinfection can be developed by the human body as a consequence of previous infections	[38]
Ethnic origin	Variation in the susceptibility to infection	[20]
Religion	Religious rules related to water contact related to disease exposure	[20,35]
Socioeconomic status	Relation to hygiene, the availability of protected water supplies and ability to cope with the disease	[39]
Migration	Population movements can modify spatial patterns of disease transmission through both introduction of the parasite or the acquisition of infection	[20,33,40]
Occupation	Work related to water increases the exposure and risk of infection (fishermen, farmers, etc.)	[34,35]
Location of house	Location of house in relation to suitability of closest water source can influence infection status	[35,41,42]
Prevention/control measures	Spatial pattern of disease transmission can be highly modified by mass treatment campaigns and successful preventive measures	[43-45]

### *Schistosoma* parasites

There are six schistosome species parasitising humans; *S. haematobium*, *S. mansoni*, *S. japonicum*, *S. intercalatum*, *S. mekongi* and *S. guineensis*. The current review focuses on the former two, which are the most widespread and affect tens of millions of people, particularly in sub-Saharan Africa [46].

Water temperature has a major influence on the prepatent period of *Schistosoma*, which is related to its abundance in the environment. However, the relevant temperature thresholds vary between different stages of parasite development. Eggs of the parasite hatch at temperatures ranging between 10°C and 30°C [18]. The period from penetration of the miracidium to initial shedding of cercariae by the snail varies with temperature between the minimum of 17 days at 30-35°C and several months at lower temperatures. Laboratory investigations found an inverse relationship between the length of the prepatent period with water temperature; the developmental null point of parasite development occurred at 14.2°C for *S. mansoni* [19] and at 15.3°C for *S. haematobium* [47]. The maximum of *S. haematobium* cercarial shedding was observed at water temperatures around 25°C [47].

Water flow velocity influences the spatial distribution of the parasite. Stagnant water contains the highest cercarial density, whereas flowing water can transport the parasite passively for considerable distances [20]. Very slow moving water with a speed of approximately 0.1 m/s is beneficial to allow the widespread dissemination of the parasite, which in turn enhances the odds of finding a suitable intermediate host snail [27,48]. Interestingly, active parasite mobility has been observed to be stimulated by various components of sweat secreted by humans [49,50].

Natural predators such as fish, and diverse carnivorous invertebrates, feed on the miracidia, and hence, they may reduce parasite abundance [20,21]. Sunlight is a particular stimulus for the release of cercariae from infected snails, whereas the number of cercariae produced is mainly influenced by the size of the snail and the water temperature [19]. Internal factors of the parasite may modify the risk of infection such as different genetic strains resulting in different pathogenicity. This could account for the observed geographical variations in severity of human schistosomiasis [22]. The efficiency of snail and human infection varies with species. For example, miracidia of *S. haematobium*, on average, need more individuals to infect their intermediate host snail than those of *S. mansoni* [19]. Additionally, intermediate host snails of *S. haematobium* are more dispersed than those of *S. mansoni*, which thus result in lower snail infection rates observed in natural freshwater bodies for *S. haematobium* [19].

### Freshwater snails

To complete a successful life cycle, *S. haematobium* is transmitted almost exclusively by snails of the genus *Bulinus*, of which, however, only a few species are susceptible [27]. Intermediate hosts of *S. mansoni* belong to the genus *Biomphalaria* [27]. Both snail genera are pulmonate snails of the Planorbidae family and live in aquatic environments [27].

Similar to the parasite, survival, fecundity and rate of reproduction of freshwater snails are sensitive to water temperature. In contrast to the parasite, snails prefer cooler conditions. Despite snails having broad tolerance ranges of their ambient temperature between day and night and over the course of a year, the most favourable range lies between 18°C and 32°C [24]. Snails of the genus *Bulinus* show a distinct peak of maximal reproduction at 25°C [25], whereas *Biomphalaria* have the highest reproduction rates at temperatures ranging from 20°C to 27°C [24,26]. The correlation between thermal regimes and snail fecundity allowed deriving a critical level of 120-179 degree hours greater than 27°C per week. When this critical level was exceeded, snails were absent from a habitat [24]. Indeed, 100% mortality was observed after exposure of snails to 36°C and above within a few days [18]. However, in sub-tropical regions of Africa, the impact of temperature on the limitation of snail distribution is only relevant for very small water bodies exposed to continuous high temperatures [23].

Regarding water flow velocity, freshwater snails have a noticeable narrow tolerance range [24] and become dislodged when flow velocity exceeds approximately 0.3 m/s [24,28]. A nearly linear, negative correlation between the density of *Biomphalaria* and water flow velocity was derived until the aforementioned limit [24,28]. It has also been shown that snails are highly dispersed along streams and irrigation schemes [51], which can result in the agglomeration of snails and parasites in downstream areas, especially during and after rains occurred. Based on the assumption that higher stream order (in the hierarchical network of topographically induced water drainage) is linked to decreased flow velocity, a positive association between stream order and *S. mansoni* infection prevalence in school-aged children has been observed in the Man region of western Côte d’Ivoire [52].

Vegetation determines habitat suitability of freshwater snails in several ways. First, the presence of aquatic vegetation is positively linked to the amount of dissolved oxygen and the consumption of carbon dioxide (CO_2_) and thereby linked to movement and reproduction of pulmonate snails [23]. Secondly, snails seek broad-leafed vegetation as surface to crawl on and deposit their egg masses, whereas the periphyton, which encrusts the submerged parts of the plant, provides the food supply for snails [23].

The nature of substratum of a water body is related to snail abundance. Firm mud rich in decaying organic matter provides a favourable habitat for snails. In contrast, clean sand, semi-liquid mud or a bottom with low organic matter does not provide a suitable snail habitat [30,34].

Water depth is related to the distribution of freshwater snails, which are generally found in shallow water near the margins of their habitats as a function of food, shelter and light conditions [29,53]. Snails are rarely found at depth in excess of 1.5-2 m. However, they are able to survive at a depth of up to 10 m, but there is little risk of transmission of schistosomiasis whenever snails are at such high depths [29].

Sudden fluctuations of water levels, such as irrigation channels operated by pump schemes, provide habitats of low suitability to establish a snail population [23]. Importantly though, snail populations are able to persist in temporary habitats through their ability to aestivate during periods of drought in sheltered spots, under vegetation, on mud or in mud crevices [23,24]. In general, compared to *Biomphalaria*, *Bulinus* are more successful to withstand periods of prolonged desiccation, surviving up to one year by burying themselves beneath the substratum [24]. During this time, development of the parasite and cercarial shedding may be suspended temporarily [54].

Rainfall modifies snail habitat conditions in manifold ways. Heavy rainfall may sweep away snail populations due to high flow velocity [24,27]. Furthermore, temporary habitats are created by enduring rainfall events and snails can establish a population either if they survived desiccation or by being passively transported to a temporary habitat with the discharge of the rain. Another relevant aspect with respect to the probability of disease transmission is that rainfall directly supports the contamination of water through washing human excreta charged with *Schistosoma* eggs into potential snail habitats [20].

Water turbidity due to a high content of suspended minerals (360 mg/l) can impact the reproduction cycle of freshwater snails through smothering egg masses preventing development and hatching of eggs. However, adult snails do not seem to be affected [23,30]. Growth of aquatic plants is limited due to high turbidity and thereby habitat conditions become less favourable [23].

With regard to water chemistry and quality, a low pH may be directly harmful to snails [27]. Indeed, the frequency of snails was found to be associated with water hardness with a preference of snails for very hard waters [23]. Maximum tolerated concentrations and lethal concentrations of certain ions for snail species have been quantified by Deschiens [31]. In general, *Bulinus* show a greater tolerance to changing chemical conditions than *Biomphalaria*. However, the latter genus was found to have higher tolerance to chloride (Cl^−^) and sodium (Na^+^) concentrations than *Bulinus* [23,31]. Industrially polluted waters were found to be unsuitable for intermediate host snails, whereas high snail abundance was found near human habitations, perhaps explained by waters polluted by human excreta that contain *Schistosoma* eggs [23].

Similar to the stimulation of cercarial shedding with daylight, snails themselves were observed to be noticeably active in sunlight [23]. Egg masses of snails are often seen in direct sunlight and are apparently unaffected [23]. Furthermore, sunlight corresponds to the flourishing of aquatic weeds, the abundance of microflora and thereby a high content of dissolved oxygen rendering the water highly suitable for snails [23,24]. In contrast, completely shaded pools provide unsuitable habitat conditions for snail proliferation [23].

There are several vertebrate and invertebrate predators that influence the abundance of snails [23]. Parasites such as leeches or trematodes and pathogens such as fungi, viruses and bacteria may be pathogenic to snails [23]. However, both predators and pathogens would mainly limit the abundance of aquatic snails [23]. Finally, the respective snail species have differing genetic predispositions that result in an intraspecific variation of the susceptibility to the miracidium of a parasite as well as its efficiency to produce cercariae [27,32].

### Humans as definitive hosts

Humans are the definitive host for *S. mansoni* and *S. haematobium*. Cercariae penetrate the intact skin of the human, transform to schistosomula, enter the blood stream, develop, pair up and start producing eggs within approximately two weeks [2]. Adult schistosome worm pairs produce eggs over the entire course of their life and part of the eggs are excreted through faeces (*S. mansoni*) or urine (*S. haematobium*). Schistosomiasis is a disease of poverty [2,55]. In African countries south of the Sahara, poverty usually goes hand-in-hand with poor hygiene and housing, limited access to clean water and improved sanitation, subsistence farming and low educational level – all of them exacerbating the risk of acquiring schistosomiasis [55]. Furthermore, low socioeconomic status is intimitely connected with the lack of a household to cope with the disease, as preventive and curative measures are out of reach.

The contamination of surface waters and their surroundings with human excreta containing *Schistosoma* eggs is an important driver of schistosomiasis transmission that is governed by human behaviour [20]. The rate of infection has been observed to be significantly higher in people living in houses without a latrine and without access to piped water [34,35,56,57]. However, water contact behaviour is the major, decisive factor related to the risk of acquiring schistosomiasis. Even when the environmental setting provides most suitable conditions for the transmission of the disease, infection does not occur, if people do not get in contact with freshwater bodies. Major activities related to infection were identified to be lack of personal hygiene, swimming, washing clothes and children playing in water [33]. Less critical activities were the washing of objects, fetching water and crossing water bodies most likely due to the relative short duration of water contact and the relatively small surface areas of the human body exposed to infested water [33].

The relationship between gender and risk of infection is equivocal and varies with the cultural background of the people [35,58,59]. In some regions, higher prevalence of infection in females could be related to the fact that water-related activities were several-fold greater for women than men [36], whereas an opposite relationship was found when men dominated the activities with exposure to water [37]. School-aged children and adolescents are usually at highest risk of infection with peak prevalences found at around 10-15 years [33]. This characteristic age-prevalence curve is due to high exposure when fetching and playing in water at this age [35] and there might be partially acquired immunity to the disease at an older age [33]. However, especially preschool-aged children and their mothers are at considerable risk due to water contact, although only relatively few studies have been conducted to further our understanding of the epidemiology and schistosomiasis in preschool-aged children and to come forward with recommendations for treatment and control that are tailored to specific settings [60-65]. Of note, the higher the transmission, the earlier the peak of infection prevalence is observed. This phenomenon has been termed the ‘peak shift’ [66]. Interestingly though, a community-based study in Côte d’Ivoire revealed a second peak at old age (>50 years) [67]. With regard to immunity, it has been shown that humans are able to develop defence mechanisms that modify the effects of exposure with increasing age [38]. Hence, this typical convex shape of the age-prevalence and age-intensity curves with respect to schistosomiasis is argued to be related to a slow acquisition of immunity to reinfection following a slow death of adult worms from early infections [33,38]. This is the basic argument that development of a schistosomiasis vaccine should be possible [68].

Furthermore, it has been observed that the acute stage of schistosomiasis was rarely found in indigenous populations, yet often in travellers to endemic areas [20]. Large water resource development projects attract migrant workers and their families to hot spots of disease transmission [20]. Semi-permanent or seasonal workers migrate to large agricultural projects for harvesting, often related to extensive irrigation schemes [33,69,70]. These people are at high risk of acquiring schistosomiasis if they enter endemic areas; however, they may (re-)introduce the parasite into controlled or non-endemic areas [20]. It has also been observed that after the construction of new rail and highway systems in West Africa, the spatial distribution of schistosomiasis changed, following these transportation routes [40]. Differences in the susceptibility to the disease with respect to ethnic origin of humans have been attributed to the immunological response influenced by different ancestral experiences with the infection [20].

Religion plays a role when respective rules govern practices that may significantly affect patterns of water use [35]. For example, ritual washing five times a day before prayer as required by male Muslims significantly affects the prevalence of *Schistosoma* infection in the respective communities [20].

A strong correlation between occupation and risk of infection is not surprising, particularly if work is related to water exposure [35]. For example, farmers and farm laborers, and especially fishermen and boatmen, showed higher *Schistosoma* prevalence rates than factory workers [34].

The location of a household in relation to the suitability of a water body to transmit the disease has shown to be highly relevant with respect to the level of prevalence [35]. Indeed, a study in Ghana found that high infection levels were clustered around ponds known to contain intermediate host snails of *S. haematobium*, whilst prevalence was low in households in close proximity to a river where the intermediate host snails were rarely found [41]. A study by Mota and Sleigh [42] concluded that the relative location of a house to snail-free or snail-colonised water sources was a key driver explaining the spatial pattern of *S. mansoni* infection in Brazil.

The use of control measures alters the prevalence and intensity of infection, and thus morbidity due to schistosomiasis, and might impact on disease transmission. Following mass treatment campaigns, the level of prevalence and morbidity decreased considerably [43,71]. However, numerous studies revealed that reinfection occurs rapidly whenever preventive measures have been neglected (see, for example [44,45]), thus not addressing the root social and ecological causes of schistosomiasis [72-75].

### Remote sensing of schistosomiasis

The first application of remote sensing to predict the probability of occurrence of human schistosomiasis using Landsat 5 Thematic Mapper (TM) data was published in 1984 for the Philippines by Cross et al. [76]. Initially, the use of remote sensing data to estimate the impact of disease casualty rates was driven by military and economic interests and only later applied for epidemiological investigations [77].

A decade after the pioneering work by Cross and colleagues, diurnal temperature differences derived from data of the National Oceanic and Atmospheric Administration-Advanced Very High Resolution Radiometer (NOAA-AVHRR) have been related to survey measurements of schistosomiasis prevalence in Egypt [78]. As thermal differences between day and night reflect regional hydrologic conditions [79], the significant inverse relationship showed well the predictive ability of remote sensing data for schistosomiasis transmission risk [78].

We were interested in more recent applications of remote sensing for schistosomiasis risk profiling. Hence, we determined the number of published studies by accessing the online library PubMed (http://www.ncbi.nlm.nih.gov/pubmed/). Our search was conducted in early February 2015 according to the terms and Boolean operators provided by the review of Simoonga et al. [11] covering the period between 1 January 1995 and 31 December 2014, complemented with a detailed focus on reviewing remote sensors, environmental variables, as well as temporal and geographical coverage of data (Table 3). Our PubMed search resulted in 93 publications, of which 31 were relevant reviews or case studies using remote sensing data for spatial modelling of schistosomiasis risk. Within the process of reviewing the literature and critically examining the cited references, the number of relevant studies increased to 37.

**Table 3:** **Overview of remote sensing data and derived environmental variables investigated for spatial analyses of schistosomiasis from 1 January 1995 to 31 December 2014**

**Satellite sensor**	**Environmental variable**	**Sensor combination**	**Geographic area**	**Temporal coverage**	**Reference data**		**Reference(s)** ^*^
					**Snail**	**Human infection**	
NOAA-AVHRR spatial resolution: 1.1 km	Diurnal temperature difference NDVI	Landsat	Egypt	Monthly time series 1990-1991	Snail occurrence, infection rate (water survey 1 km distance to population survey)	Survey prevalence (rural health units)	[80]
	LST, NDVI	SRTM	Tanzania	Monthly time series 1985-1998		Survey prevalence (school)	[81]
	LST, NDVI		Ethiopia – East Africa	Annual + seasonal composites 1992-1996		Survey prevalence (town/village) – 5 km buffer	[82]
	LST, NDVI		Ethiopia	Annual + seasonal composites 1992-1996	Snail occurrence	Survey prevalence (town/village) – 5 km buffer	[83]
	LST, NDVI		Cameroon	1985-1998		Survey prevalence (district – stratified at school level)	[84]
	LST, NDVI	Landsat GTOPO30	Uganda	Not indicated		Survey prevalence, infection intensity (school/village)	[85]
	LST, NDVI		Kenya, Ethiopia, Uganda	Annual + seasonal composites 1992-1995	Snail occurrence	CEGET/WHO atlas – 5 km buffer	[86]
	Land cover	MODIS, Landsat, METEOSAT, GTOPO30	Côte d’Ivoire	1992/1993		Survey prevalence (school)	[87,88]
	LST, NDVI	GTOPO30	Tanzania	1982-1998		Survey prevalence (school)	[89]
	LST, NDVI	GTOPO30	East Africa	1982-2000		Survey prevalence (school)	[90]
	Land cover	GTOPO30	Côte d’Ivoire	1992/1993		Survey prevalence (school)	[91]
	LST, NDVI		Burkina Faso, Mali, Niger	Not indicated		Survey prevalence (school)	[92]
	LST, NDVI		Nigeria	2001-2002		Survey prevalence (school)	[93]
	LST, NDVI		Zambia	1992-1995	Snail occurrence, cercarial shedding	Survey prevalence, infection intensity (school/village)	[94]
	LST, NDVI		East Africa	1982-2000		Survey prevalence (school)	[95]
	LST, NDVI		Tanzania	Not indicated		Survey prevalence (school)	[96]
	LST, NDVI		Burkina Faso, Mali	1982-1998		Survey prevalence (school)	[97]
	NDVI		Sudan	Not indicated		Survey prevalence (village)	[98]
	LST, NDVI	SRTM	Sierra Leone	Not indicated		Survey prevalence (school)	[99,100]
	LST, NDVI		Ghana	Not indicated		Survey prevalence (school)	[101]
MODIS spatial resolution: 1 km, 500 m, 250 m	LST, NDVI	NOAA-AVHRR, Landsat, METEOSAT, GTOPO30	Côte d’Ivoire	Monthly time series January + November 2002		Survey prevalence (school)	[87,88]
	LST, NDVI		Uganda	Annual + seasonal composites 2000-2003	Snail occurrence	Survey prevalence (school)	[102]
	LST, NDVI		Uganda	Annual + seasonal composites 2000-2003	Snail occurrence	Survey prevalence (school)	[103]
	LST, NDVI, Land cover	GTOPO30	West Africa	2000-2008		Survey prevalence (school)	[104]
	LST, NDVI Land cover	GTOPO30	East Africa	2000-2009		Survey prevalence (school/community)	[105,106]
	LST, NDVI	METEOSAT, SRTM	Côte d’Ivoire	Not indicated		Survey prevalence (school)	[107]
Landsat TM/ETM + spatial resolution: 30 m, 60 m	Spectral bands: (blue(1), red(3), mir(5), thermal(6)) NDVI Tasseled cap: brightness, greenness, wetness	NOAA-AVHRR	Egypt	May 1990	Number, distribution, infection rate	Survey prevalence (rural health units)	[80]
	Water body map	NOAA-AVHRR, GTOPO30	Uganda	March 2000		Survey prevalence, infection intensity (school/village)	[85]
	Settlements, roads, rivers	NOAA-AVHRR, MODIS, METEOSAT, GTOPO30	Côte d’Ivoire	January + November 2002		Survey prevalence (school)	[87,88]
	NDMSI (normalized difference moisture stress index)	Ikonos, SRTM	Kenya	June 1986 + January 2003	Snail/shell occurrence		[51]
		ᅟ					
Ikonos spatial resolution: 1 m	Land cover	Landsat, SRTM	Kenya	March	Snail/shell occurrence		[41,51,108]
METEOSAT spatial resolution: 8 km	Rainfall estimates	NOAA-AVHRR, MODIS, Landsat, GTOPO30	Côte d’Ivoire	September 2001–August 2002		Survey prevalence (school)	[87,88]
	Rainfall estimates	MODIS, SRTM	Côte d’Ivoire	Not indicated		Survey prevalence (school)	[107]
SRTM spatial resolution: 90 m	Altitude	NOAA-AVHRR	Tanzania	February 2000		Survey prevalence (school)	[81]
	Altitude, slope, stream order, catchment		Côte d’Ivoire	February 2000		Survey prevalence (school)	[52,107]
	Altitude, slope, drainage network	Landsat, Ikonos	Kenya	February 2000	Snail/shell occurrence		[51]
	Altitude	NOAA-AVHRR	Sierra Leone	February 2000		Survey prevalence (school)	[99]
GTOPO30 spatial resolution: 30 sec (ca. 1 km)	Altitude	NOAA-AVHRR, Landsat	Uganda	1994-1997		Survey prevalence, Infection intensity (school/village)	[85]
	Altitude	NOAA-AVHRR, MODIS, METEOSAT, Landsat	Côte d’Ivoire	1994-1997			[87,88]
	Altitude	NOAA-AVHRR	Tanzania	1994-1997		Survey prevalence (school)	[89]
	Altitude	NOAA-AVHRR	East Africa	1994-1997		Survey prevalence (school)	[90]
	Altitude	NOAA-AVHRR	East Africa	1994-1997		Survey prevalence (school)	[91]
	Altitude	MODIS	Côte d’Ivoire	1994-1997		Survey prevalence (school)	[104]
	Altitude	MODIS	East Africa	1994-1997		Survey prevalence (school/community)	[106]

*References are listed manifold if the study investigated data from more than one remote sensor.

The potential of remote sensing and its combination with geographical information system (GIS)-based spatial analyses for schistosomiasis risk profiling can be summarised by the ability (i) to determine the geographical limit of disease distribution due to ecological constraints of disease transmission; (ii) to further investigate the context of disease ecology and epidemiology through its spatial relation; (iii) to support prevention, surveillance and control through prioritising areas of disease risk; and (iv) to provide early warning for areas where disease transmission could become established [4,9,11,80,109-112]. However, most studies had an integrative focus combining some of the different analytical steps from spatial disease delineation to early warning mentioned above. The most frequently used remote sensing data are from the NOAA-AVHRR and later the Moderate Resolution Imaging Spectroradiometer (MODIS) with ground resolutions of 1.1 km and 250 m, respectively. Only few studies have used high resolution data from Landsat 5 TM (30 m) and very high resolution remote sensing data (1 m) have solely been investigated for one study site in Kenya [41,51,108].

Topographic information from either Shuttle Radar Topography Mission (SRTM) data or the global digital elevation model with 30-arc second resolution (GTOPO30) were added in most studies as predictor variable. The environmental parameters most commonly used were the NDVI and land surface temperature (LST), hypothesised to represent surrogate measures of environmental moisture and temperature, respectively [82]. The availability of AVHRR and MODIS data free of charge and the online access to pre-processed imagery boosted studies that investigated these data [16]. However, as already stated by Herbreteau et al. [16], there are many other vegetation- [113] or moisture-related indices [114] not directly accessible due to their more complex nature, which are rarely used for health studies. Several studies (see, for example [97,99,100]) have used spatial information of perennial water bodies and river networks from the GeoNetwork platform provided by the Food and Agriculture Organization (FAO) of the United Nations [115]. However, the acquisition dates back to the 1990s and it lacks information on temporal dynamics. Availability of more recent data would be highly relevant to monitor environmental changes such as construction of dam lakes or irrigation schemes [1]. Satellite remote sensing provides data and methodological procedures to map and monitor water bodies and other disease relevant parameters such as water temperature, turbidity or vegetation coverage [12].

Reference data for the spatial analysis with remote sensing data were mostly point data, either of human infection prevalence most frequently geo-located at schools, or snail occurrence located in sampled water bodies (Table 3). Overall, snail data were very rarely available and most analyses were based on infection of school-aged children surveyed. In most studies, infection of school-aged children has been determined using parasitological examinations of stool and urine examination and microscopy, whilst only few studies were built upon prevalence data based on morbidity questionnaires (see, for example [96]). Epidemiological data of human infection or snail sampling often had a temporal mismatch of several years between the sampling and the acquisition of remote sensing data (see, for example [95,104]). However, this has been considered as negligible drawback, since schistosomiasis is a chronic disease with a life-span of adult worms being typically several years in the absence of treatment interventions [20]. Therefore, spatial variability in long-term synoptic environmental factors is hypothesised to have more influence on transmission success and infection patterns than seasonal variability in a location [95]. When environmental variables such as NDVI or LST are used to predict the risk of schistosomiasis, “in effect, one is predicting the environmental requirements for a particular snail species (infected with a particular parasite species) – and not the human parasitic infection *per se*” [11]. An analysis of remote sensing data with respect to snail abundance and disease prevalence showed that snail distribution generally corresponded to the prediction model of schistosomiasis prevalence, however, the best model of snail distribution showed different ranges of temperature than found in schistosomiasis prevalence models [82,83]. One remaining challenge to further improve remote sensing and GIS-based risk profiling is to account for the spatial mismatch between the school-based measurement of human infection and the water body, where disease transmission occurs and remote sensing variables have the potential to provide ecologically relevant measurements to characterise habitat conditions of disease-related parasites and intermediate host snails.

Environmental analyses using remote sensing data provide the opportunity for a deeper understanding of the process underlying broad-scale patterns of schistosomiasis distribution and can help to potentially improve our knowledge of schistosomiasis infection ecology [9]. To give an example, a study by Raso et al. [87] found that – besides age, sex and socioeconomic status – rainfall pattern and elevation significantly explained the geographical variation of *S. mansoni* distribution in the mountainous Man region, western Côte d’Ivoire. For the same region, Beck-Wörner et al. [52] found a significant correlation for stream order of the closest river, the water catchment and altitude. However, the observed significant relationship between *Schistosoma* prevalence among school-aged schildren and elevation was not apparent in a risk model for sub-continental West Africa [97]. For sub-continental East Africa, a negative correlation resulted from the distance to freshwater body and elevation with respect to the distribution of *S. mansoni* infection intensity [90]. This shows that predictor variables and resulting models are specific to the reference data, the scale of observation and the geography of the study site. A reasonable impact of different ecological zones on predictor performance and model outcome was first established by Brooker et al. [81] in Tanzania, a phenomenon that should be kept in mind when using remote sensing data for modelling disease risk.

Remote sensing and GIS have proven to be useful for planning and implementing disease intervention and control programmes by excluding areas where schistosomiasis is unlikely to be a public health problem and modelling priority areas of increased transmission risk [9,81]. Clements et al. [92] predicted regions with a probability of *Schistosoma* infection in the school-aged population greater than 50% in Burkina Faso, Mali and Niger, which calls for mass treatment campaigns according to guidelines put forth by the World Health Organization (WHO) [116]. Estimates of the number of school-aged children who are at particularly high risk have been predicted for West Africa [104] and East Africa [105], and for two countries; namely Tanzania [81] and Nigeria [117]. Additionally, expectable programme costs emphasising large-scale treatment administration were calculated based on model predictions. Estimated treatment costs were higher if data were aggregated on a provincial level compared to the national level, which might be explained by large spatial heterogeneities of disease risk at the sub-national level. This indicates that the issue of scale must be considered in spatially explicit risk profiling [118].

Many studies focused on modelling the risk of polyparasitic helminth infections, such as *S. mansoni* and soil-transmitted helminths, with the objective to enhance cost-effectiveness of integrated control approaches (see, for example, [88,100,119]). Mass treatment campaigns, however, often lack sustainability, because specific freshwater bodies might act as high transmission zones if they are (re-)contaminated by even a single untreated individual. This consideration suggests the importance of water-site factors to achieve a shift from morbidity to transmission control and local elimination [120-122]. In this regard, remote sensing data hold promise to monitor the presence and dynamics of water-sites and further characteristics such as water surface temperature and other environmental variables.

### Linkage between remote sensing and disease ecology in space

The review of risk factors that influence schistosomiasis transmission ecology (see Table 2) and previous applications of remote sensing data for schistosomiasis risk profiling (see Table 3) were further synthesised and condensed and are presented in Table 4. This overview is structured according to the steps of the parasite life cycle and (i) shows the potential contribution of remote sensing data for schistosomiasis risk profiling and (ii) indicates the meaning of remote sensing measurements with respect to the ecological relevance for disease transmission. In the context of modelling schistosomiasis risk, remote sensing variables can provide either direct measurements of the feature of interest, which are, for example, the measure of LST, water persistence or flow velocity or vegetation coverage along water sites. These variables are derived from respective remote sensing metrics such as land surface emissivity or surface reflectance at appropriate wavelengths according to their spectral properties. They can directly reflect habitat conditions and provide information about the potential impact on the ecology of disease transmission. On the other hand, remote sensing data provide proxy variables, where the remotely sensed measurement is not representing the respective measure influencing disease transmission but being indirectly linked to the requested information. For example, based on remote sensing data, the slope of a land surface can be measured from topographical modelling, however, the relevant information to be drawn from this proxy measure would be water flow velocity as the decisive criterion for profiling risk of disease transmission. Due to an additional step of modelling information to ecological indicators, the potential sources of errors that affect the data may increase.

**Table 4:** **Synthesis of remote sensing contribution for schistosomiasis risk profiling**

	**Factor impacting on disease transmission**	**Remote sensing (proxy) variable**	**Ecological impact**
**Parasite**	Water body	Near and middle infrared reflectance	Hatching of eggs; infection of snail and human host
	Water temperature	Thermal infrared (emissivity)	Length of prepatent period; activity, survival and infection rate
	Water flow velocity	Topography: slope angle, curvature	Determination of maximal cercarial density; passive transport of parasite
	Predators	NA	Reduction of parasite population
	Sunlight	Shaded habitats (tree coverage)	Reduction of parasite population
	Pathogenicity	NA	Severity of disease in humans
	Species	NA	Different efficiency of snail infection
**Snail**	Water body	Near and middle infrared reflectance	Fundamental habitat of snail to maintain a population
	Water temperature	Thermal infrared (emissivity)	Fecundity, mortality and rate of reproduction
	Water flow velocity	Topography: slope angle, curvature	Determination of snail density; passive transport of snail; food availability
	Vegetation	Visible and near infrared reflectance	Food supply; surface for oviposition; increase of dissolved oxygen
	Substratum	NA	Abundance of snails in water body
	Water depth	Visible and near infrared reflectance	Abundance of snails in water body
	Stability of water level	Temporal dynamic of water body	Abundance of snails in water body
	Rainfall	Cloud thickness and temperature	Creation of temporary snail habitats; modification of water flow velocity; supports contamination of water
	Turbidity	Visible and near infrared reflectance	Reproduction cycle of snails
	Water chemistry/quality	NA	Abundance of snails in water body
	Sunlight	Shaded habitats (tree coverage)	Abundance and activity of snails
	Predators, parasites and pathogens	NA	Reduction of snail population
	Species	NA	Susceptibility to parasite and cercarial productivity
**Human**	Water contact behaviour	NA	Exposure to parasite infested water; contamination of surface waters
	Gender	NA	Determinant of water contact activities (culturally variable)
	Age	NA	Related to degree of exposure and level of immunity
	Immunity	NA	Resistance to reinfection can be developed following previous infections
	Ethnic origin	NA	Susceptibility to infection
	Religion	NA	Religious practices may affect patterns of water use
	Socioeconomic status	NA	Standard of hygiene; access to protected water supply; ability to cope with disease
	Migration	NA	Modification of spatial distribution of disease
	Occupation	NA	Work related to water increases exposure
	Location of the house	Settlement mapping	Exposure of population to potential disease transmission sites
	Prevention/control measures	NA	Modification of spatial pattern of disease transmission

Table 4 illustrates that there are risk factors governing schistosomiasis transmission that can potentially be measured by remote sensing data and others that cannot. Specifically, parasite- and snail-related risk factors are well addressed by remote sensing measurements. However, it shows also very clearly that remote sensing has its natural limitations and cannot detect factors such as intrinsic factors related to parasites, snails and humans as well as some chemical and biological aspects. Thus, remotely sensed environmental measurements can contribute substantially to characterise the habitat conditions of parasites and snails and can moreover detect human settlements and their spatial relation to suitable habitat conditions. However, against this background, it has to be kept in mind that remote sensing-based approaches for disease risk profiling have gaps of information to be filled by other disciplines.

## Review; conclusion

In our view, the issue of schistosomiasis risk profiling has not been addressed with sufficient depth by interdisciplinary groups of epidemiologists and remote sensors. In this review, we made an attempt to bridge the two strands of scientific inquiry, based on specific professional expertises and experience. Taking up this task, the present work has synthesised an informed linkage between remote sensing measurements of surface conditions and ecological processes that govern the transmission of schistosomiasis. Our synthesis deepens the understanding of this interdisciplinary linkage and thereby allows quantification of remote sensing contribution for overall disease risk estimation. Our review suggests that the potential of remote sensing data for schistosomiasis risk profiling is greater than explored to date. Indeed, there is a multitude of remote sensing data and variables other than NDVI and LST available that need to be investigated to specifically address ecological conditions relevant for schistosomiasis transmission. Freely available remote sensing data sources to achieve these investigations are for example the newest Landsat 8 pre-processed data products (see, http://landsat.usgs.gov/CDR_ECV.php) with spatial resolution of 30 m, or 20 m data from the Sentinel 2 mission (http://www.esa.int/Our_Activities/Observing_the_Earth/Copernicus/Sentinel-2), which is scheduled to be launched in May 2015. Our review also emphasises that the scale of observation, geographical area and variables used impact the outcome of schistosomiasis risk models, and hence demands for a tailored approach. One remaining challenge to further improve remote sensing and GIS-based risk mapping and prediction is to account for the spatial mismatch between remote sensing measurements of potential schistosomiasis-related parasite and snail habitats and school- or community-based measurements of human infection, which has not been considered in any of the reviewed studies. Especially with regard to the unfolding agenda of elimination of schistosomiasis and other helminthiases [122-124], thus going beyond morbidity control, sites where schistosomiasis transmission potentially occurs need to be specifically addressed. A better integration of remote sensing data in epidemiology holds promise to detect and characterise potential disease transmission sites and thereby support new integrated prevention and control strategies to combat schistosomiasis.

## Abbreviations

DALY: Disability-adjusted life year
FAO: Food and Agriculture Organization
GIS: Geographical information system
GTOPO30: Global digital elevation model with 30-arc second resolution
LST: Land surface temperature
MODIS: Moderate-Resolution Imaging Spectroradiometer
NDVI: Normalized difference vegetation index
NOAA-AVHRR: National Oceanic and Atmospheric Administration-Advanced Very High Resolution Radiometer
SRTM: Shuttle Radar Topography Mission
WHO: World Health Organization
